# Supplementary material for: The genome sequence of the Red-barred Tortrix moth, Ditula angustiorana (Haworth, 1811)
Source: Wellcome Open Res. 2025 Apr 22;10:202. [Version 1] doi: 10.12688/wellcomeopenres.23994.1 (PMC12770897; doi:10.12688/wellcomeopenres.23994.1)
Supplement: Peer review history

# Reviewer report: Reviewer response for version 1

**DOI:** 10.21956/wellcomeopenres.26471.r139440

**Published:** 2026-01-05

**Authors:** Qing-Song Zhou, Jing-Yi Zhang

**Affiliations:** 1 Chinese Academy of Sciences, Beijing, China; 2 Institute of Zoology Chinese Academy of Sciences (Ringgold ID: 53024), Chaoyang, Beijing, China

This article presents a high-quality chromosome-level genome assembly of *Ditula angustiorana* using PacBio HiFi and Hi-C data. with high contiguity (as reflected by a high N50) and completeness (as indicated by high BUSCO scores). The assembly demonstrates excellent contiguity and completeness, representing a valuable resource for lepidopteran genomics. The technical approach is robust and well-executed.

 However, it is better to provide additional background information on *D. angustiorana*, including its ecological and economic relevance, biodiversity context, and taxonomic history. Such context would help readers to understanding the significance of this genomic resource.

 Additionlly, while the current assembly is already of high quality, the inclusion of Illumina short-read data for polishing and RNA-seq data for genome annotation would further enhance the accuracy and utility of this reference genome. The authors are encouraged to consider incorporating these data in future updates or supplementary analyses.

Are sufficient details of methods and materials provided to allow replication by others?

Yes

Is the rationale for creating the dataset(s) clearly described?

Yes

Are the datasets clearly presented in a useable and accessible format?

Yes

Are the protocols appropriate and is the work technically sound?

Yes

Reviewer Expertise:

biodiversity, genomics, entomology

We confirm that we have read this submission and believe that we have an appropriate level of expertise to confirm that it is of an acceptable scientific standard.

# Reviewer report: Reviewer response for version 1

**DOI:** 10.21956/wellcomeopenres.26471.r139445

**Published:** 2025-12-01

**Authors:** Hasin Ullah

**Affiliations:** 1 Northwest University, Xi'an, China

The article presents the genome assembly of *Ditula angustiorana*, a species in the family Tortricidae, sequenced as part of the Darwin Tree of Life Project. The genome sequencing provides valuable insight into the genetic makeup of this insect species, with a genome size of 468.36 megabases and chromosomally complete scaffolds for 29 autosomes and sex chromosomes. The assembly also includes the mitochondrial genome, showcasing a thorough effort to document the species' genomic landscape.

 The methodology section is well-detailed, explaining the use of PacBio HiFi long reads for sequencing and Hi-C sequencing for scaffolding the chromosomal structure. The Hi-Fi reads and Hi-C data were successfully combined to generate an accurate assembly, with quality metrics showing a QV of 64.5 and a BUSCO gene completeness of 98.4%. These quality benchmarks demonstrate a high-quality assembly.

 Additionally, the curation process, which involved visual inspection and removal of contaminants and misjoins, ensured the assembly's accuracy. Specific issues like haplotypic duplications were addressed, and corrections improved the scaffold N50, a critical metric for assembly quality. This shows that the authors are meticulous in their approach and committed to ensuring a high-quality product.

Are sufficient details of methods and materials provided to allow replication by others?

Yes

Is the rationale for creating the dataset(s) clearly described?

Yes

Are the datasets clearly presented in a useable and accessible format?

Yes

Are the protocols appropriate and is the work technically sound?

Yes

Reviewer Expertise:

Now I am working on insect morphology, but my previous work was on genomics.

I confirm that I have read this submission and believe that I have an appropriate level of expertise to confirm that it is of an acceptable scientific standard.
